# Stereoscopic view synthesis with progressive structure reconstruction and scene constraints

**DOI:** 10.1371/journal.pone.0279249

**Published:** 2022-12-19

**Authors:** Wei Liu, Liyan Ma, Bo Qiu, Mingyue Cui

**Affiliations:** 1 Electromechanic Engineering College, Nanyang Normal University, Nanyang, Henan, China; 2 Computer Engineering and Science College, Shanghai University, Shanghai, China; 3 Electronic and Information Engineering College, Hebei University of Technology, Tianjin, China; Kingston University, UNITED KINGDOM

## Abstract

Depth image-based rendering (DIBR) is an important technology in the process of 2D-to-3D conversion. It uses texture images and related depth maps to render virtual views. While there are still some challenging problems in the current DIBR systems, such as disocclusion occurrences. Inpainting methods based on deep learning have recently shown significant improvements and generated plausible images. However, most of these methods may not deal well with the disocclusion holes in the synthesized views, because on the one hand they only treat this issue as generative inpainting after 3D warping, rather than following the full DIBR processing procedures. While on the other hand the distributions of holes on the virtual views are always around the transition regions of foreground and background, which makes them more difficult to distinguish without special constraints. Motivated by these observations, this paper proposes a novel learning-based method for stereoscopic view synthesis, in which the disocclusion regions are restored by a progressive structure reconstruction strategy instead of direct texture inpainting. Additionally, some special cues in the synthesized scenes are further exploited as constraints for the network to alleviate hallucinated structure mixtures among different layers. Extensive empirical evaluations and comparisons validate the strengths of the proposed approach and demonstrate that the model is more suitable for stereoscopic synthesis in the 2D-to-3D conversion applications.

## Introduction

Recently, 3D videos have become more and more popular compared to traditional 2D videos, because they offer richer representation of real-world scenes. The success of the 3D industry has led to a growing demand for 3D content. However, it is not easy to make content directly in some appropriate 3D formats, and the cost of making 3D videos is still comparatively high. 2D-to-3D conversion technology can add 3D effects to a large number of existing 2D format media data, so it provides a practical way to solve the bottleneck of 3D content for 3D media applications.

Researchers have given significant attention to the depth image based rendering (DIBR) technology, which synthesizes virtual views at different viewpoints with a 3D warping process from the image-plus-depth data format [1]. When the depth map is available, DIBR systems can generate any number of views without multi-camera systems; thus the equipment cost of 3D cinema systems is reduced. Additionally, the transmission bandwidth required by the image-plus-depth data format can be reduced compared with two color images by at least 33%. For these advantages, DIBR technology has been recognized as a promising tool. However, the quality of synthesized virtual views may be affected by the compression of texture image and depth map coding during transmission. To overcome this, studies on coding distortion elimination [2, 3] and joint code bit allocation [4] were made for synthesized image quality improvement. Besides that, an inherent problem with 3D warping algorithms is that a given pixel does not necessarily exist in both views. Therefore, the background regions occluded by the foreground object in the reference view may be exposed in the virtual view due to sharp horizontal changes among different depth layers in the warping process. In order to solve this problem and obtain high-quality 3D effects, these holes need to be filled.

To deal with these occlusions, one solution is to rely on more complex multi-dimensional data representations such as layer depth image (LDI) allowing additional depth and color values to be stored for occluded pixels in the original view. This additional data provides the necessary information to fill the occluded area in the rendered virtual view. However, this entails increasing the overhead complexity of the system. On the other hand, removing occlusion can be realized by pre-filtering the depth map to reduce the discontinuity of depth data in such a way that disocclusions decrease, and then post-processing the warped image to replace the missing area with some color information. This is a common pipeline which current DIBR systems almost always follow [5].

In the depth pre-filtering approach, holes are diminished before 3D warping rather than being filled later. Early studies preferred various low-pass filters to smooth the depth image, as described in [6]. The main disadvantage is that they tend to introduce additional geometric distortion or artifacts in disocclusion areas. To solve this problem, several methods have been proposed, including asymmetric smoothing [7, 8], scene structure, or content-related adaptive filters [9, 10]. These methods aimed to retain stronger smoothing in specially restricted regions rather than the whole images; thus excess smoothing can be avoided in the non-hole regions. However, this type of method reduces the 3D effects as the depth map is smoothed. Moreover, the introduction of blurry regions around the large holes makes them unsuitable for situations in which the virtual view is far away from the reference view. With the post-processing approach, the holes are filled either by texture replication or by structure continuation after DIBR with auxiliary information around disocclusion regions [11–13]. These methods can largely maintain the accuracy of the propagated texture structures while avoiding some local artifacts. Nonetheless, the foreground texture may need to be sampled to fill the holes. In order to alleviate this problem, some improved methods employ depth or foreground/background information as reasonable constraints on depth-based view synthesis to exclude the foreground textures in the filling process. These methods extract low-level features from uncorrupted regions to match and paste patches. However, they do not synthesize plausible content in complex scenes where non-repetitive patterns appear. Furthermore, they have high computational cost due to their iterative nature [14].

In contrast to the early traditional approaches, many deep learning-based methods have recently been developed to solve these problems. Geometry-based view synthesis methods [15–18] have been proposed to synthesize novel views without depth maps. These approaches add geometry constraints to preserve consistency between input views and the synthesized view. Unfortunately, cues such as view transformation information or additional camera pose are not required in the application of 2D-to-3D conversion. Other works adopted a strategy that combined monocular depth estimation with the DIBR process in one CNN framework, such as Deep3D [19] and also previous work [20], where a probabilistic selection layer was proposed to model the rendering process in a differentiable way so that it could be trained together with a depth map prediction network. Additionally, some image-content-based methods [21–23] have formulated the view synthesis task as a mapping from input views to the target view directly without depending on explicit geometrical supervision. The rationale behind is that the collective power of massive training data provides regularizations on the learned-view transformations.

Unlike these approaches, this paper describes how our method handles the image-plus-depth data formats where the depth maps given to systems may be captured by active approaches with range devices or generated by a 2D-to-3D converter from different sources [24]. Therefore, the accuracy of the given depth maps may well be an issue and might further affect the quality of the synthesized view greatly, being more difficult to deal with than visual inpainting in general. Research based on deep learning in this field is still limited. Some studies build networks to handle the disocclusion holes appearing after the DIBR procedure. In particular, they regard the disocclusion problem as a generative image inpainting challenge and use learning-based inpainting techniques [25, 26] to restore the occlusion regions in the warped views. In our opinion, for these methods the main focus is on texture inpainting rather than modeling the full DIBR processing procedures, which may not deal with the disocclusions well in complicated scenes without enough constraints. In this paper, we propose a novel network where the disocclusion regions are restored by a progressive structure reconstruction strategy across all traditional stereoscopic synthesis processing pipelines. In this way, more visual scene features can be exploited as prior knowledge to improve recovery performance. In addition, although current learning-based inpainting methods achieve plausible results, they still suffer from texture artifacts and structure preservation problems from limited information in stereoscopic synthesis. These failures appear especially in the transition regions of different layers. We can explain this by the fact that most current methods assume that scene structure and layer information can be implicitly learned by CNNs without any further supervision; thus, no additional information is provided to the model. However, in 3D warped views most holes are distributed around the layer transition regions, which increases the difficulty of distinguishing the layer boundaries clearly without explicit priors. To overcome these limitations, some special constraints of the synthesized scenes are further exploited in our network to alleviate hallucinated structure mixtures in the warped views.

We can summarize our contributions as follows:

- A novel learning-based network framework for stereoscopic view synthesis is proposed, in which the disocclusion regions are restored by a progressive feature reconstruction strategy so that more edge and structure cues can be generated gradually to help in describing the virtual scene for better solutions to the disocclusion hole filling task.
- Two scene constraints, especially effective for stereoscopic synthesis, are further exploited for our network. In this way, our method achieves good performance on virtual view quality, and is more suitable than previous approaches for the 2D-to-3D conversion application.

The rest of the paper is organized as follows. Firstly, the technical scheme of proposed approach will be introduced in depth. Then the experimental results are reported and discussed, and finally some concluding remarks are given.

## The proposed approach

### Framework of the proposed scheme

**Fig 1:**
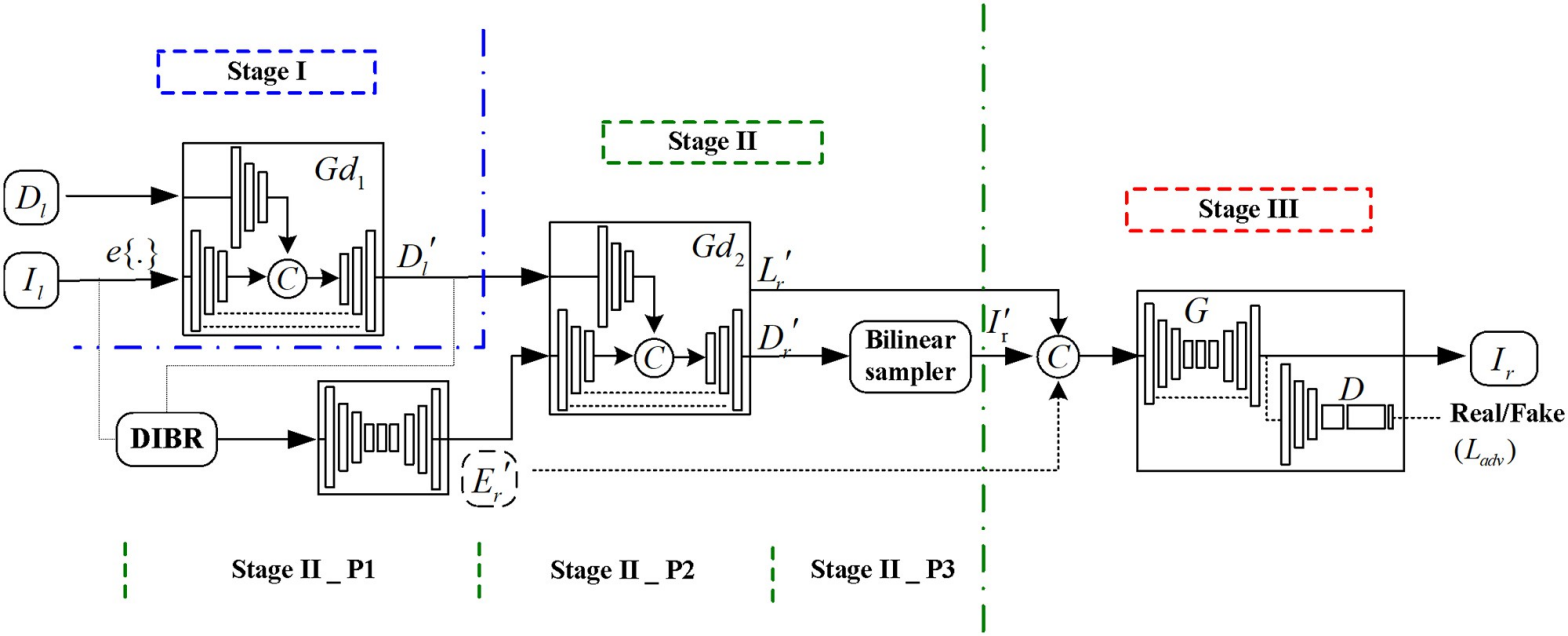
Framework of the proposed learning-based stereoscopic synthesis approach.

Given an input view *I*_*l*_ and the corresponding depth map *D*_*l*_, our goal is to estimate the image at the novel view. For notational convenience, in the following the estimation of the right virtual view *I*_*r*_ will be made more explicit. Formally, we can write this as:

$$\begin{array}{c} I_{r}=f\left(I_{l},D_{l}\right) \end{array}$$ (1)

where *f* is a function that defines the relationship between the input view and the novel view. Therefore, we propose to learn about this relationship. This relationship is usually complex as it requires finding connections between different views and collecting appropriate position information from a depth map. Inaccuracies such as noise in the depth map may further add to the complexity of this relationship. To deal with these problems, in this paper we propose multi-stage modeling to comply with the traditional stereoscopic view synthesis procedures and divide our system into there different estimation stages as shown in Fig 1, including:

- Stage I: Joint guided filtering before 3D warping
- Stage II: Progressive reconstruction of scene features on the novel view
- Stage III: Image refinement with a residual learning-based generative adversarial network (GAN)

Stage II further contains the following three phases:

- Stage II_P1: Layer aware scene edge recovery module
- Stage II_P2: Joint guided scene depth/layer recovery module
- Stage II_P3: Initial novel view prediction

In this way, our proposed method follows the complete DIBR processing pipelines and is better suited to 2D-to-3D conversion applications than existing similar technologies based on deep learning. We will discuss each stage at length in the following.

#### Joint guided filtering before 3D warping

In our system, we first use a joint guided filtering network *Gd*_1_ to estimate the optimized depth map $D_{l}^{\prime }$, which aligns the depth discontinuities to color discontinuities in texture image *I*_*l*_:

$$\begin{array}{c} D_{l}^{\prime }=Gd_{1}(D_{l},\left\{I_{l},\Delta \left(I_{l}\right)\right\}) \end{array}$$ (2)

where Δ(.) represents the Laplacian operator, *D*_*l*_ is the target input, and edge map Δ(*I*_*l*_) and texture image *I*_*l*_ are concatenated together as the guidance inputs. The estimated depth map $D_{l}^{\prime }$ is then used to warp the input image to the novel view:

$$\begin{array}{c} \left\{I_{w},D_{w},M\right\}=w(I_{l},D_{l}^{\prime }) \end{array}$$ (3)

where *w*(., .) represents the 3D warping operator. *M* is the mask indicating the different regions, a binarized matrix in which 1 represents the missing region and 0 represents the background, and which is consistent with the hole distributions of the warped image *I*_*w*_ and warped depth map *D*_*w*_.

#### Progressive reconstruction of scene features on the novel view

In the second stage, the novel view is coarsely estimated through progressive scene feature reconstructions, which can be further divided into three phases. In each phase, a specific sub-task is tackled:

$$\begin{array}{c} \left\{ \begin{array}{c} P1:E_{r}^{\prime }=En(\Delta (I_{w}⊙M))\\ P2:\left\{D_{r}^{\prime },L_{r}^{\prime }\right\}=Gd_{2}\left(D_{l}^{\prime },E_{r}^{\prime }\right)\\ P3:I_{r}^{\prime }=b\left(I_{l},D_{r}^{\prime }\right) \end{array}\right. \end{array}$$ (4)

where *P*1, *P*2, and *P*3 represent the three phases, ⊙ is the pixelwise multiplication, and *b*(., .) is a bilinear interpolation operator used to realize backward warping. In the first phase, an edge estimation network *E*_*n*_ is adopted to recover the scene edges on the novel view. The hole-filled edge map $E_{r}^{\prime }$ is then used to guide the estimation of the depth map $D_{r}^{\prime }$ on the novel view by another joint guided filter *Gd*_2_, which has a similar network structure to *Dd*_1_ in the first stage. In particular, the layer structure $L_{r}^{\prime }$, which is trained by smoothed ground-truth virtual view image with method [27] and would be concatenated in the final refinement stage, can simultaneously also be learned and estimated by *Gd*2 as another output. By this multiple modality, the features extracted in the generation of $L_{r}^{\prime }$ encourage the disocclusion hole filling of $D_{r}^{\prime }$ taken in different layers. It is in turn beneficial to make the estimation of the depth map $D_{r}^{\prime }$ layer-aware. Finally, we perform backward warping with bilinear interpolation *b*(., .) to synthesis the coarse initial novel view $I_{r}^{\prime }$ by sampling the original view *I*_*l*_ based on the depth map $D_{r}^{\prime }$.

#### Image refinement with a residual learning-based GAN

In the third stage, a GAN-based refinement network *G* exploits all the previous predictions to generate a high-quality novel view with restored scene features and realistic texture details as following, which generates the final optimized virtual view *I*_*r*_:

$$\begin{array}{c} I_{r}=G(I_{r}^{\prime },L_{r}^{\prime },E_{r}^{\prime },M) \end{array}$$ (5)

In this way, the latent features in the stereoscopic synthesis can be fused into the network naturally, helping prevent irrelevant foreground pixels from being warped to the holes and achieving disocclusion hole filling in the warped view more effectively than existing generative inpainting technologies based on deep learning.

### Hole filling with progressive scene feature reconstruction and constraints

**Fig 2:**
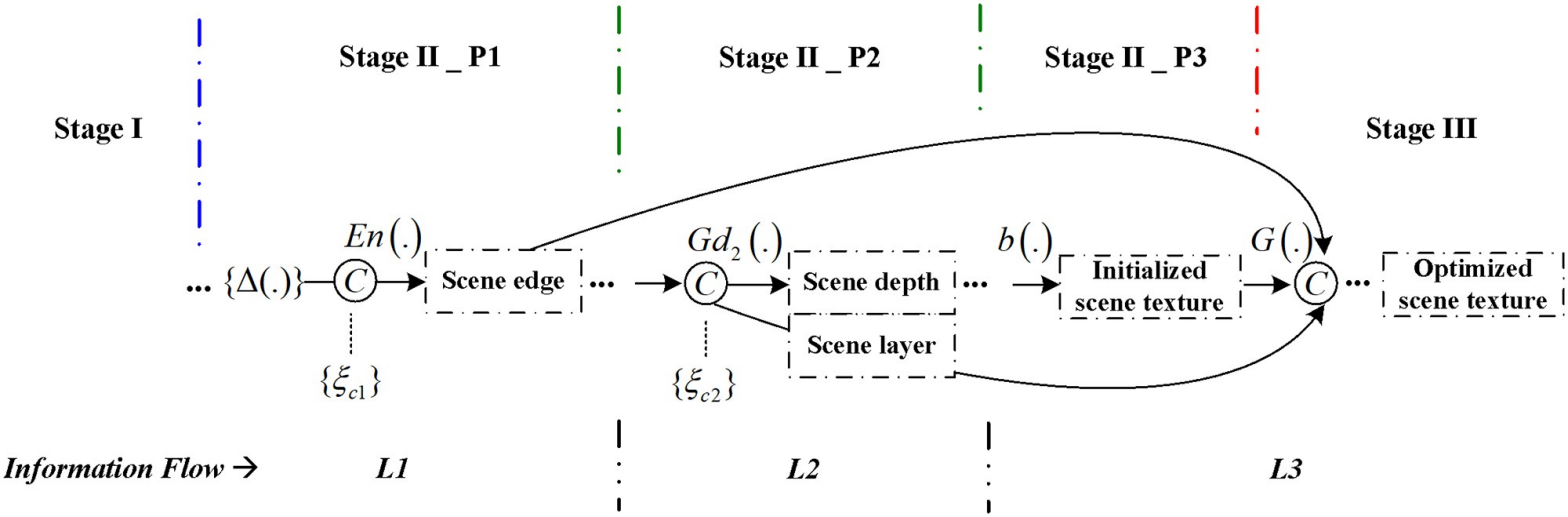
Information flow of progressive scene feature reconstruction on the novel view with constraints.

In this paper we propose multi-stage modeling of stereoscopic synthesis, which can also be formulated as a curriculum-learning problem. As presented in Fig 2, the information flow displays our novel three-level progressive reconstruction procedure method adopted for stereoscopic-view inpainting, following a strategy we have named “line first, structure next, texture last”. The details are summarized as follows:

- The first level (*L1*) is scene edge recovery, where the scene edges with holes are initially restored by our edge estimation network *E*_*n*_ in the first phase *P1* of Stage II. This philosophy of “line first, color next” was previously introduced by the recent approach of [28], where the edges were recovered as intermediate information to support the completion network. In our work, on the other hand, these constraints are not sufficient to fill the disoccluded holes due to the problem that these holes are all along the transition regions between the foreground and background content. To better deal with these issues, an explicit edge constraint *ξ*_*c*1_ is added, and it will be discussed in depth below.
- The second level (*L2*) is scene structure recovery, mainly realized in the second phase *P2* of Stage II. For this level, complete scene depth and scene layer maps $\left\{D_{r}^{\prime },L_{r}^{\prime }\right\}$ are both recovered based on the extracted edge feature $E_{r}^{\prime }$ on *L1*. These scene cues have removed high-frequency texture details while retaining low-frequency structures, so they can be regarded as meaningful intermediate scene structures to represent the global structures of the virtual scene on the novel view. Besides this, they will be used to guide the texture reconstruction at the last level. In this way, our network can focus on recovering global structures without being disturbed by irrelevant texture information. As with *L1*, in this part another constraint *ξ*_*c*2_ is added to enhance the scene structure recovery for the novel view.
- After reconstructing the missing structures on *L2*, a texture generator is used to synthesize high-frequency details on the third level (*L3*), which mainly includes the third phase *P3* of Stage II as well as Stage III. First, initialized scene textures are generated by backward warping based on the reconstructed depth map $D_{r}^{\prime }$ on *L2*. Then, final optimized scene textures are predicted by a GAN-based sub-network with enhanced scene edge and structure priors from both *L1* and *L2*.

In addition to the novel three-level progressive reconstruction procedure scheme described above, in this section we further exploit two other scene constraints {*ξ*_*c*1_, *ξ*_*c*2_} that are especially effective for the stereoscopic synthesis in this paper. In fact, these two constraints are always to be regarded as important scene cues and have been effectively used in some traditional DIBR approaches [5, 6]. While most of the current deep-learning based methods assume that these constraints can be implicitly learned by CNNs without any further supervision, and therefore do not provide any additional information to the model. In this study we found that it is not easy for the existing deep learning-based generative inpainting methods to learn these cues well. So we added {*ξ*_*c*1_, *ξ*_*c*2_} as priors on different processing stages of our network and realized constraint enhancements in a progressive way. The experimental results demonstrate that the improvements are obvious and hence this strategy is effective for the disocclusion hole filling in the warped view.

The first constraint *ξ*_*c*1_ is essentially an extracted layer boundary map, which we use to boost the scene edge recovery in particular for stereoscopic synthesis. As discussed above, the disocclusion holes are mainly distributed around the transition regions of different layers, so the acquisition of accurate layer boundaries is important for the subsequent image completion process. While previous deep learning-based generative inpainting methods do not have explicit constraints to address these issues. Here we proposed constraint *ξ*_*c*1_ on *L1* to overcome limitations, as shown in Fig 2. For stereoscopic synthesis, the layer boundary that distinguishes the foreground and background is just to one side of the hole contour. For the right virtual view it is on the left side while for the left view it is on the right side. The edge recovery module is not facilitated to learn this cue implicitly without any further supervision. Combining this cue as an extra constraint to our network is an effective way to help fill the disocclusion holes with more accurate structure boundaries. Based on this observation, the layer boundary map *ξ*_*c*1_ is defined as:

$$\begin{array}{c} \xi_{c1}\left(p\right)=\{\begin{array}{cc} sgn(p), & if\;D_{w}\left(p_{l}\right)>D_{w}\left(p_{r}\right)\\ 1-sgn(p), & otherwise \end{array},for\;p\in \Delta \left(M\right) \end{array}$$ (6)

where Δ(.) again represents the Laplacian operator, *p*_*l*_ and *p*_*r*_ are the left and right pixels respectively of *p*, and *sgn* is a pre-defined symbolic function. In our work, for the right virtual view, *sgn*(*p*) = 1, and for the left view, *sgn*(*p*) = 0. It is then added to *E*_*n*_ so as to generate more plausible layer boundaries in $E_{r}^{\prime }$. In this way, our method can find the layer boundaries according to the directional characteristics provided by the warped depth map *D*_*w*_.

Similarly, the second constraint *ξ*_*c*2_ on *L2* is proposed to enhance structure reconstruction with a coarsely generated layer distribution map. For the depth prediction of the disocclusion region, we assume that the missing region and its surrounding background content belong to the same physical surface, so they should have similar depth values. Based on this cue, we apply a fast directional nearest interpolation approach to achieve initial hole filling of depth values on the warped depth map *D*_*w*_, which is then further used as a layer distribution map to guide the scene layer reconstruction within the novel view. More precisely, the processed depth value in pixel *p* for each horizontal line across a hole of *D*_*w*_ is defined as:

$$\begin{array}{c} d\left(p\right)=D_{w}\left(p_{0}\right),\;where\;p_{0}\in \left(\Delta \left(M\right)-\xi_{c1}\right)\cap D_{w} \end{array}$$ (7)

In this way, the disocclusion regions are filled with depth values from the nearest background pixel in the line, which is useful for marking the hidden membership of the disocclusion holes. The layer distribution map *ξ*_*c*2_ can now be expressed as follows:

$$\begin{array}{c} \xi_{c2}\left(p\right)=D_{w}\left(p\right)⊙\left(1-M\right)+d\left(p\right)⊙M \end{array}$$ (8)

With this form, this constraint can help guarantee the missing region and its surrounding background content belong to the same layer. In our network, it is concatenated as a guidance of *Gd*2 to improve the prediction of the missing region in the subsequent depth/layer reconstruction network.

Considering these two scene constraints, the information flow of Stage II, shown logically in Fig 2 and already formulated in Eq (4), can be updated as:

$$\begin{array}{c} \left\{ \begin{array}{c} P1:E_{r}^{\prime }=En(\Delta \left(I_{w}⊙M\right),\xi_{c1})\\ P2:\left\{D_{r}^{\prime },L_{r}^{\prime }\right\}=Gd_{2}\left(D_{l}^{\prime },\left\{E_{r}^{\prime },\xi_{c2}\right\}\right)\\ P3:I_{r}^{\prime }=b\left(I_{l},D_{r}^{\prime }\right)\;\; \end{array}\right. \end{array}$$ (9)

### Network architecture

The overall architecture of the proposed stereoscopic synthesis network is shown in Fig 1 above. To complete such a process, some key components are required, including two sequential joint filter block-based CNNs {*Gd*1, *Gd*2}, an edge recovery net *En*, and a residual learning-based GAN {*G*, *D*}. We elaborate on these components one by one as follows.

In this work, {*Gd*1, *Gd*2} share a common deep joint filtering network architecture with different input configurations. This network architecture consists of three major components, each of which is a three-layer network [29]. Two branches, *CNN*_*T*_ and *CNN*_*G*_, act first as feature extractors to determine informative features from both target and guidance images. These features are then concatenated as inputs of *CNN*_*F*_ in the feature fusion part to transfer common structures and reconstruct the filtered output. This model has been indicated to be more effective than the straightforward implementation, which concatenates the target and guidance images together directly.

Our edge generator *En* comes from the previous work [28], and consists of encoders that down-sample twice, followed by eight residual blocks and decoders that up-sample images back to the original size. Dilated convolutions with a dilation factor of two are used instead of regular convolutions in the residual layers. In experiments we found that the pre-trained parameters in different databases can realize satisfactory results for our edge map recovery, so these parameters are used to initialize network weights for transfer learning during training.

Our last stage is constructed to further refine the coarsely predicted novel-view $I_{r}^{\prime }$, and which follows an adversarial model; that is, the stage consists of a generator/discriminator pair {*G*, *D*}. The generator *G* has an encoder that down-samples the image twice, followed by eight residual layers, and a decoder that up-samples the image back to the original resolution. Gated dilated convolutions are used in the residual layers. For the discriminator *D*, we use *PatchGAN* architecture, which determines whether or not overlapping image patches are real. All the convolutional layers employ a stride of 2 × 2 pixels to decrease the image resolution while increasing the number of output filters. Some improvements are made in this part to make this adversarial model more suitable for stereoscopic synthesis. Generator *G* has a residual learning structure, that includes short-range and long-range residual connections. Short-range residual connection refers to the local shortcut connection in each residual layer, while long-range residual connection refers to the connection directly linking the input and output of the module. This architecture has some advantages. On the one hand, this part of the model is only a sub-network in the whole framework, so gradients can be directly propagated through the long-range residual connections to upper layers to speed up training for all network components. On the other hand, initial priors from the predicted novel-view $I_{r}^{\prime }$ are provided for newly exposed hole regions. This is different from the original works of generative image inpainting, where no additional information is given for the missing regions. Therefore, our network can be trained more effectively by long-range residual connection while at the same time avoiding hallucination of pixels.

### Loss functions and training strategy

All layers of our proposed framework are differentiable, and thus end-to-end training with a single loss at the end comparing the synthesized novel view with the ground truth view is possible. However, to effectively integrate all the modules, in this work a step-by-step training strategy is employed. And it has been proved to be an effective way for network training in various kinds of applications, usually with coarse-to-fine or complex GAN-based network architectures [30–32] similar to ours. More precisely, we divide the training procedures into two sub-phases.

In the first phase, the cascaded CNN sub-modules {*Gd*1, *Gd*2} and *En* are trained as a whole with complex reconstruction losses. The multiple modality of *Gd*2 in our network is realized by further adding nodes to predict the structure layer labels in addition to the depth nodes in the final layer. Formally, the loss functions during the first-phase network training are composed of two parts:

$$\begin{array}{c} L_{rec}=E(\|I_{r}^{\prime }-I_{gt}\left\|\right) \end{array}$$ (10)

$$\begin{array}{c} L_{lay}=E(\|L_{r}^{\prime }-L_{gt}\left\|\right) \end{array}$$ (11)

where *E*‖.‖ is the Euclidean norm, *I*_*gt*_ denotes the corresponding ground truth of $I_{r}^{\prime }$, and *L*_*gt*_ is the edge-preserved smooth result [27] of *I*_*gt*_. Note that one may train the depth estimator by minimizing the error between the estimated $D_{r}^{\prime }$ and ground truth depths *D*_*gt*_, since the backward warping *b*(., .) in Eq (4) is totally differentiable. Based on this observation, an additional auxiliary loss is proposed to help optimize the learning process if the ground truth depths *D*_*gt*_ in databases are available. Then, Eq (10) can be updated as:

$$\begin{array}{c} L_{rec}=\lambda_{i}E(\|I_{r}^{\prime }-I_{gt}\left\|\right)+\lambda_{d}E(\|D_{r}^{\prime }-D_{gt}\left\|\right) \end{array}$$ (12)

where λ_*i*_ and λ_*d*_ are the weights for different terms in the loss. This part of our framework aims to optimize the CNNs to estimate good virtual views rather than depth maps. So the master branch loss in Eq (10) takes the most responsibility, while the auxiliary term in Eq (12) only helps optimize the learning process. During training, auxiliary intermediate loss can be inserted to guarantee the learned parameters carrying their corresponding physical meanings as well.

Because the reconstruction loss penalizes only the pixel-wise error, it cannot ensure that the data distribution is similar to that of the natural images. Therefore, it can easily lead to blurry inpainting results. This can be alleviated by imposing an adversarial loss, which is based on a GAN [33]. So as our proposed framework shown in Fig 1, the refinement network is trained with a residual learning-based GAN, which consists of generator loss along with adversarial loss. The overall loss function is:

$$\begin{array}{c} L_{total}=L_{G}+\alpha_{adv}L_{adv} \end{array}$$ (13)

where *α*_*adv*_ is a constant chosen to adjust the weight between generator loss and adversarial loss, and is set to 0.1 in this paper.

The adversarial loss *L*_*adv*_ is defined as:

$$\begin{array}{c} L_{adv}=\underset{D}{\text{max}}\;E[\text{log}\;D(I_{gt},M)+\text{log}(1-D(I_{r},M))] \end{array}$$ (14)

while the generator loss further integrates a content loss, perceptual loss, and style loss, and which is expressed as:

$$\begin{array}{c} L_{G}=\alpha_{con}L_{con}+\alpha_{perc}L_{perc}+\alpha_{style}L_{style} \end{array}$$ (15)

where *α*_*con*_, *α*_*perc*_, *α*_*style*_ are the loss term weights. For our experiments, we choose *α*_*con*_ = 1, *α*_*perc*_ = 0.1 and *α*_*style*_ = 250. The content loss is defined as the Euclidean distance between the predicted novel view *I*_*r*_ and the ground truth *I*_*gt*_:

$$\begin{array}{c} L_{con}=E(\|I_{r}-I_{gt}\left\|\right) \end{array}$$ (16)

and perceptual loss *L*_*perc*_ and style loss *L*_*style*_ are formulated respectively as follows:

$$\begin{array}{c} L_{perc}=E[\sum_{i}\frac{1}{N_{i}}\|\phi_{i}(I_{r})-\phi_{i}(I_{gt})\|] \end{array}$$ (17)

$$\begin{array}{c} L_{style}=E_{j}[\|Gr_{j}^{\phi }(I_{r})-Gr_{j}^{\phi }(I_{gt})\|] \end{array}$$ (18)

where *ϕ*_*i*_ is the activation map of the *i*′th layer of a pre-trained network. In our work, the *ϕ*_*i*_ corresponds to the activation maps from layers of the VGG-19 network pre-trained on the *ImageNet* dataset, which are also used to compute style loss. $Gr_{j}^{\phi }$ is a *Gram* matrix constructed from activation map *ϕ*_*j*_.

During the course of the optimization, the standard optimization of a neural network is turned into a min-max optimization problem in which at each iteration the discriminator networks are jointly updated with the generator network. By considering the overall loss function in Eq (13), the optimization becomes:

$$\begin{array}{c} \underset{G}{\text{min}}\;\underset{D}{\text{max}}\;E[L_{G}+\alpha_{adv}\;\text{log}\;D(I_{gt},M)+\alpha_{adv}\;\text{log}(1-D(I_{r},M))] \end{array}$$ (19)

where the generator and the discriminator networks are written as *G* and *D*, respectively. Let us denote the parameters of the generator network *G* by *θ*_*G*_. In the standard stochastic gradient descent, the above min-max optimization then means that, for training *G*, we take the gradient of the loss function with respect to *θ*_*G*_ and update the parameters so that the value of the loss function decreases. The gradient is:

$$\begin{array}{c} E[\nabla_{\theta_{G}}L_{G}+\alpha_{adv}\nabla_{\theta_{G}}\;\text{log}(1-D(I_{r},M))] \end{array}$$ (20)

We update the discriminator network *D* similarly, except we take the update in the opposite direction so that the loss increases.

## Experimental results and discussions

### Experimental setup

We use the *Tensorflow* platform combined with the edge generator module running on *PyTorch*, to implement the proposed method on a standard desktop with a 32 GB *NVIDIA Quadro* GPU and a batch size of 16. The model is optimized using an *Adam* optimizer with *β*_1_ = 0.0005 and *β*_2_ = 0.9 respectively. For each set, the depth maps and texture images of two different views {*I*_*l*_, *D*_*l*_, *I*_*r*_, *D*_*r*_} are provided. Training is done with 54640 sub-images of resolution 256 × 256, half generated from 20 data sets in the *Middlebury stereo 2014* database and the other half from data sets in the *KITTI 2015* database. In this way, our training sets include multiple scene sets, which can be used to better evaluate the performance of the proposed network. Data augmentation is performed on the fly, applying random transform to the training data.

To test our proposed network, different datasets are used. One part is the remaining data sets in the *Middlebury* and *KITTI* databases, which are not contained in the training data. The other part is from the *NYU Detpth* database, where indoor scenes are recorded by both the RGB and Depth cameras. Experimental results are discussed in two subsections to follow. The first is organized to show the experimental details of the proposed network framework and the effects of the additional scene constraints. In the second subsection, our results are comprehensively compared with other related works and evaluated with quantitative and subjective criteria.

### Analysis of experimental details

#### The effect of a multi-stage modeling network framework

In this subsection, processing details of our proposed network will be displayed in different experiments. As discussed in the section on the technical scheme of the proposed approach above, our multi-stage modeling network framework can refine the initial depth maps to ensure the accuracy of the depth edge to the best degree possible. Test sets in the *KITTI 2015* database have only sparse depth information rather than accurate dense depth maps. So an initial experiment on this database would mainly be analyzed to show the strong pre-processing performance of our proposed method.

**Fig 3:**
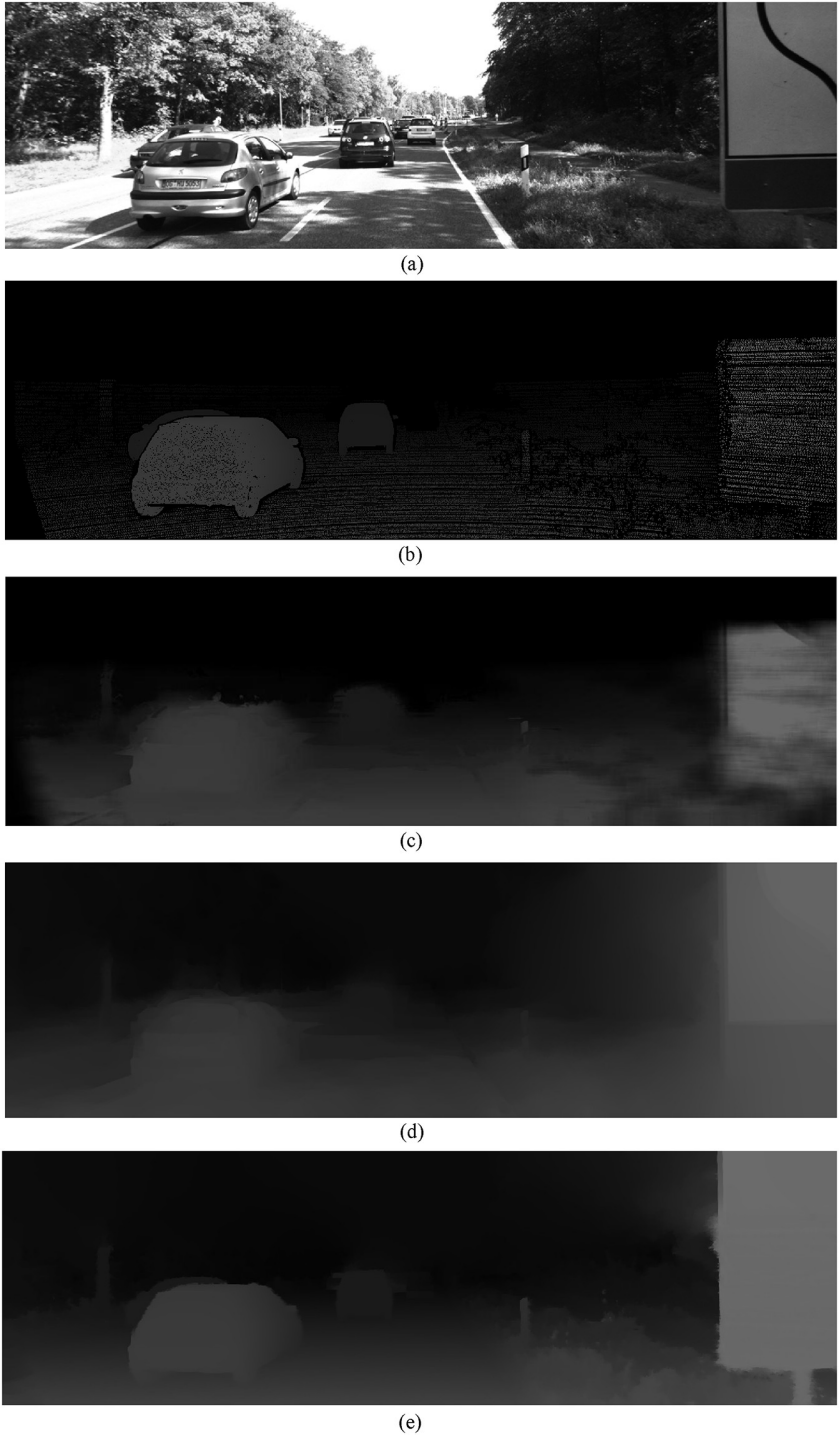
Comparison of pre-processed depth maps using different methods on data sets from *KITTI 2015*. A: Texture image, B: original sparse depth map, dense depth map generated by C: the colorization scheme, D: domain transform filter, E: our proposed network.

In this experiment, intermediate results of our proposed network are compared with depth maps pre-processed by a few other methods. As shown in Fig 3B, an original depth map in *KITTI 2015* just contains only projected sparse depth samples from *LiDaR* scan, not totally matched with the corresponding texture image in Fig 3A. So depth map background interpolation is necessary. An effective colorization scheme [34] is adopted in Fig 3C, and it can be seen that a dense depth map is generated clearly. However, some details of the scene structure are not presented perfectly in the experimental result, such as the boundaries of the car in the depth map, which are not precisely consistent with the ones in Fig 3A. In Fig 3D, another domain transform filter-based method [35] is carried out for comparison. We notice that for this method the improvement is still limited, and in some aspects other new problems such as over-smoothing may be observed. Different from these two traditional methods, the approach proposed in this paper is a data-driven solution combining both texture image and the initial depth cues in Fig 3C to the network. In this way, hallucinated cues learned from databases and local cues learned from initial depth maps are considered together. So our method can do more optimization to the initial depth map, displaying the richest depth layers and the most accurate layer boundaries as shown in Fig 3E. Thus, through this experiment, it can be seen that our method can effectively pre-process initial depth maps from different sources for a relatively stable quality, which is important for a robust DIBR system.

#### The effects of additional scene constraints

For our method, in addition to the common features often used in generative image inpainting approaches, two additional scene constraints are exploited for the stereoscopic synthesis. In this part, an experiment on the data set *Storage* from *Middlebury* is carried out to further analyze the effects of these constraints.

**Fig 4:**
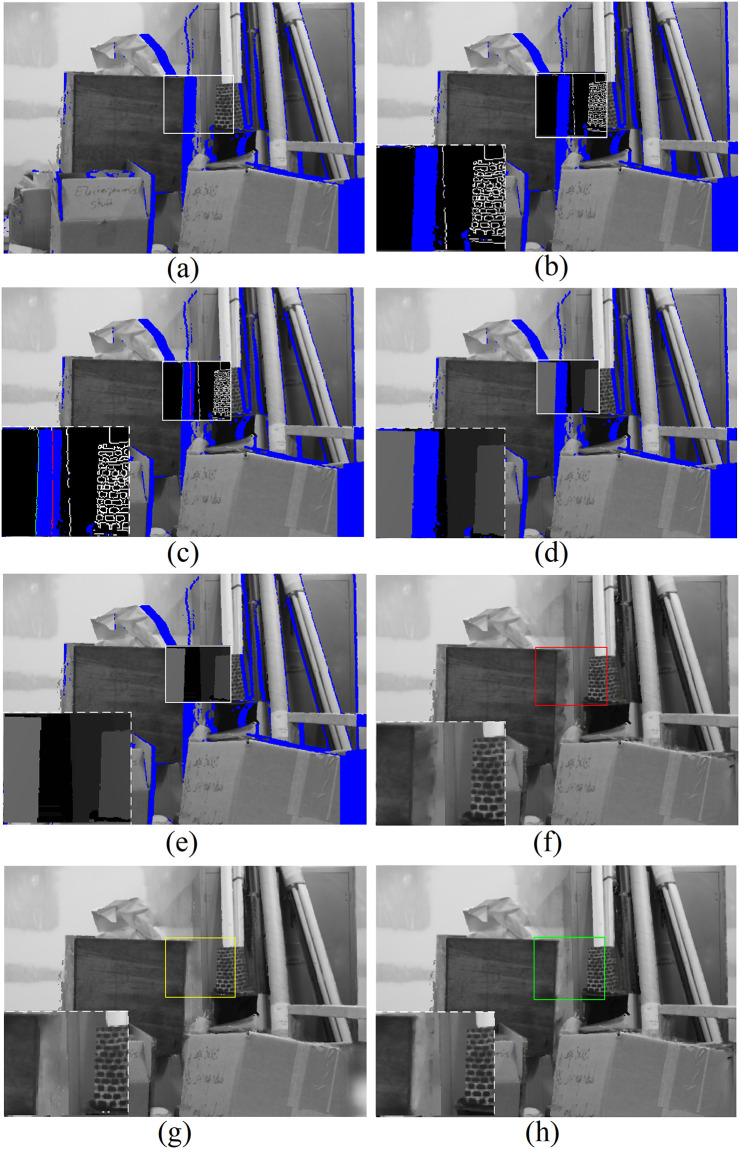
Intermediate experimental results with additional scene constraints on *Middlebury* datasets (*Storage*). A: Warped texture image with ROI, B: warped edge map in a ROI, C: recovered edge map with constraint *ξ*_*c*1_ in the ROI, D: original warped depth map in the ROI, E: re-initialized depth map with constraint *ξ*_*c*2_ in the ROI, F: generated virtual visual image without any additional scene constraints, G: with only constraint *ξ*_*c*1_, H: with both scene constraints.

In this experiment, to avoid influences by other factors the original accurate depth map *D*_*l*_ in the database was directly set as one input at *StageII* instead of the intermediate depth maps $D_{l}^{\prime }$ from *StageI*. The original image of the test set is regarded as a left view. For the right view, the areas of the newly exposed holes marked in blue are located along the right side of foreground objects as shown in Fig 4A. Obviously, the structure information around the holes belongs to the foreground and background separately and these are always different. It is hard for generative inpainting methods to reconstruct the holes using only background texture cues without reasonable guidance. The white rectangle area in Fig 4A is set as a *region of interest* (ROI); in the following we mainly focus on the ROI of the intermediate experimental results. The warped edge map displayed in the ROI of Fig 4B is used as a primarily guided constraint by our network, where the disocclusion regions are also marked in blue. From Fig 4C, it can be seen that the primarily restored parts for an edge map in the ROI are line structures. So it is much easier to deal with disocclusion holes for a warped edge map than a warped image, for which a more complicated texture recovery process would be carried out. It should also be noticed that the line marked in red was reconstructed by the edge recovery module for the disocclusion parts, while the line marked in green was restored by our proposed constraint *ξ*_*c*1_. Evidently, the line marked in green plays a more important role in helping the the network distinguish layer boundaries. Intermediate experimental results for the scene constraint *ξ*_*c*2_ are shown in Fig 4D and 4E, from which we can see that the restored depth information of the disocclusion parts in the ROI came totally from background. Thus the network can be further guided to fill the holes with the related cues just from the parts with similar depth distributions. The effects of these scene constraints for the generated virtual visual images are represented in Fig 4G and 4H respectively. Compared to the result in Fig 4F, we can see the great improvements they have accomplished in image visual quality.

#### Quantitative analysis of ablation study

We further conduct ablation studies quantitatively for testing. The quantitative comparison in Table 1 indicates how the proposed strategies in our scheme, including additional scene constraints in the progressive reconstruction procedure and training with a residual learning-based GAN for image refinement, considerably improve the performance of our network. Furthermore, this experiment demonstrates that scene structure restoration for coarse disocclusion hole region prediction is more crucial than the subsequent refinement stage in our proposed scheme.

**Table 1:** Ablation study results.

Methods	PSNR(dB)	SSIM
Full	31.05	0.9267
Full w/o additional scene constraints	28.73	0.9041
Full w/o residual learning-based GAN	29.89	0.9129

### Virtual view evaluation

In this part, we subjectively and objectively compare our methods with some other view synthesis methods on the test sets, including:

- Recent deep learning-based image inpainting methods. In our experiments, Edge Connect (*EC*) [28], Structure Flow (*SF*) [36], and Gated Convolutions (*GC*) [37], which treat the hole-filling problem as generative image inpainting with the processing scheme described in [38], are compared with ours.
- The proposed method with only the scene constraint *ξ*_*c*1_ (*Propose1*), and with all scene constraints (*Propose2*).

**Fig 5:**
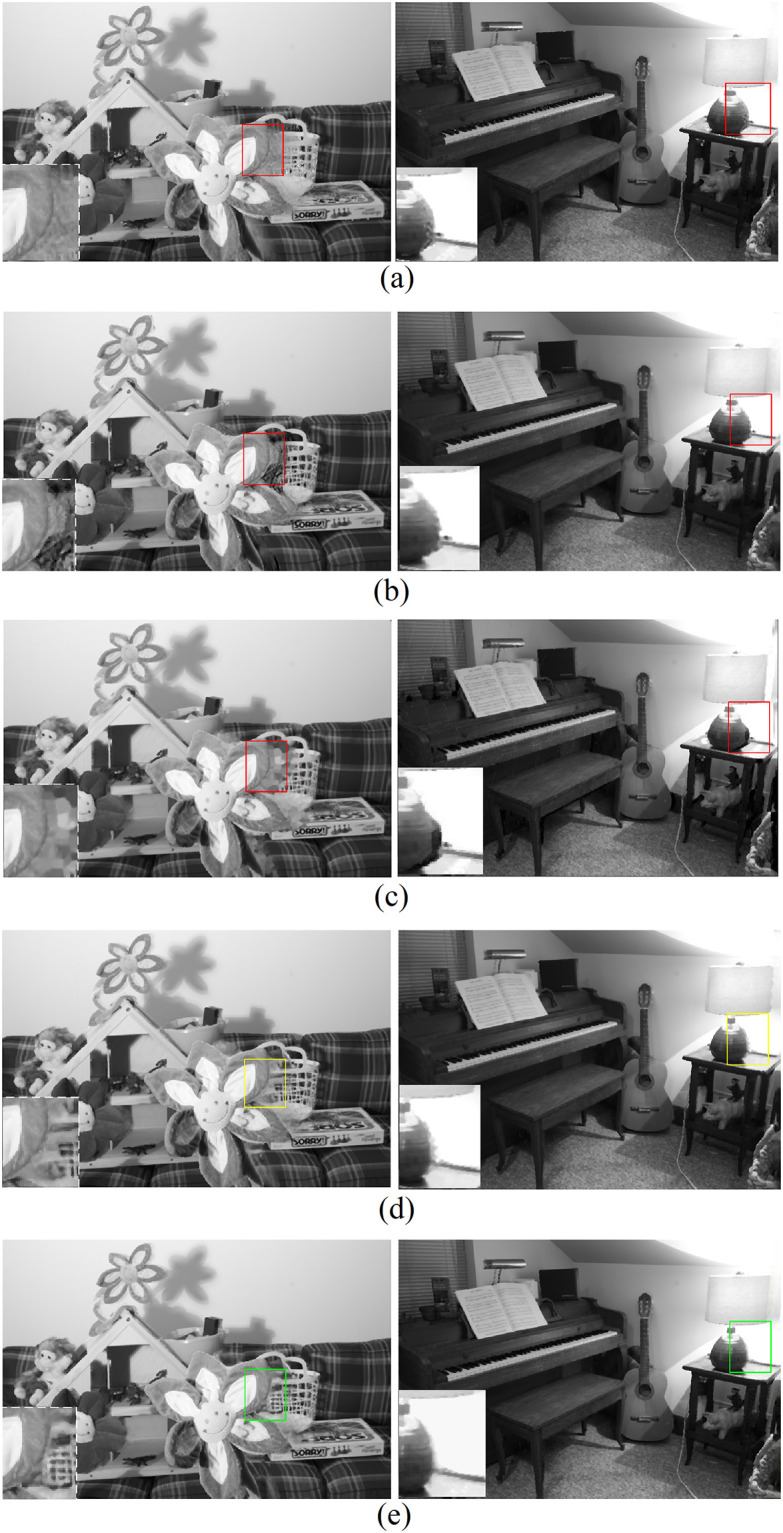
Virtual view images using *Middlebury* datasets including (from left to right) *Flower* and *Piano*, from top to bottom, A: *EC* B: *SF* C: *GC* D: *Propose1* E: *Propose2*.

**Fig 6:**
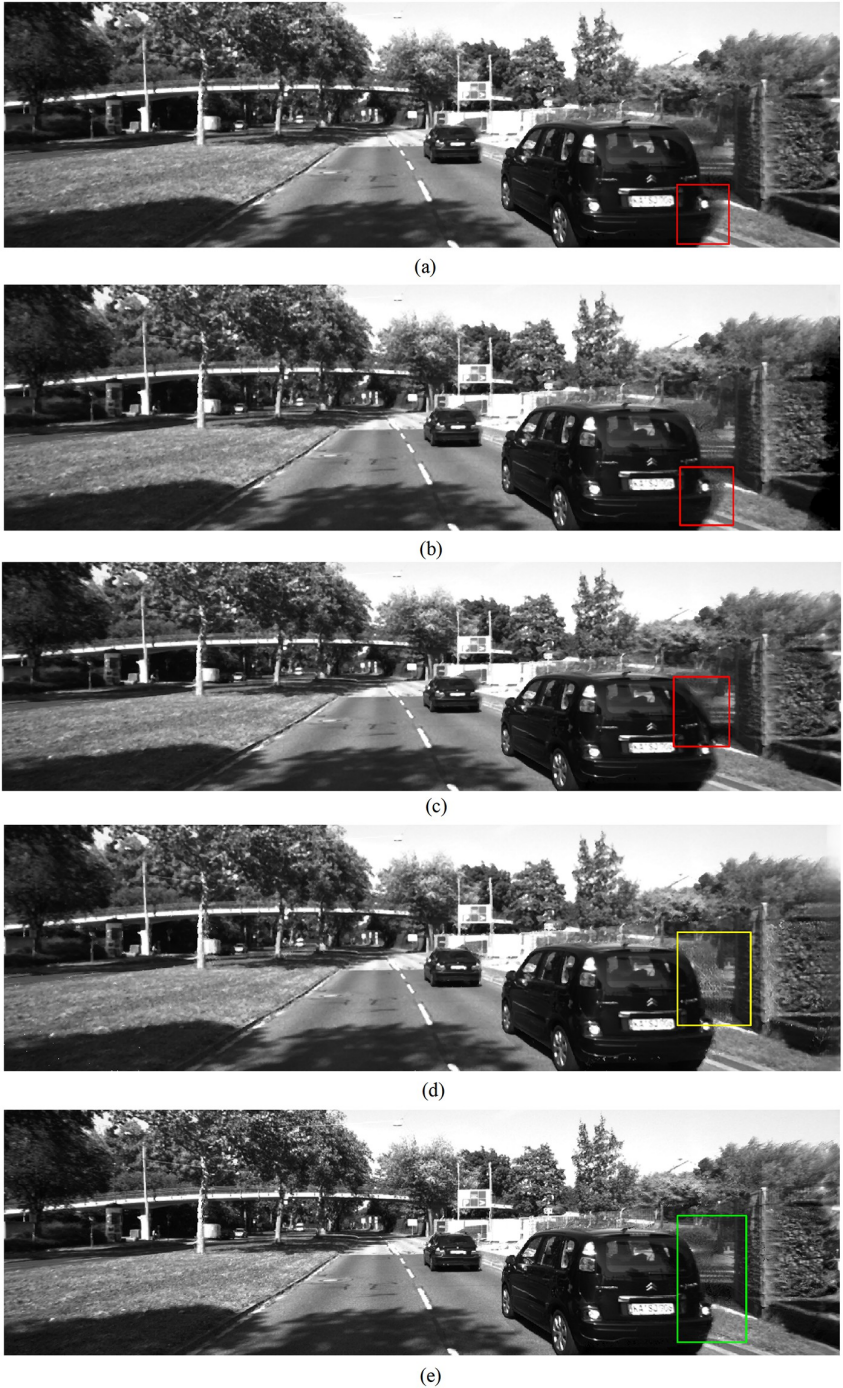
Virtual view images using *KITTI 2015* datasets, from top to bottom, A: *EC* B: *SF* C: *GC* D: *Propose1* E: *Propose2*.

**Fig 7:**
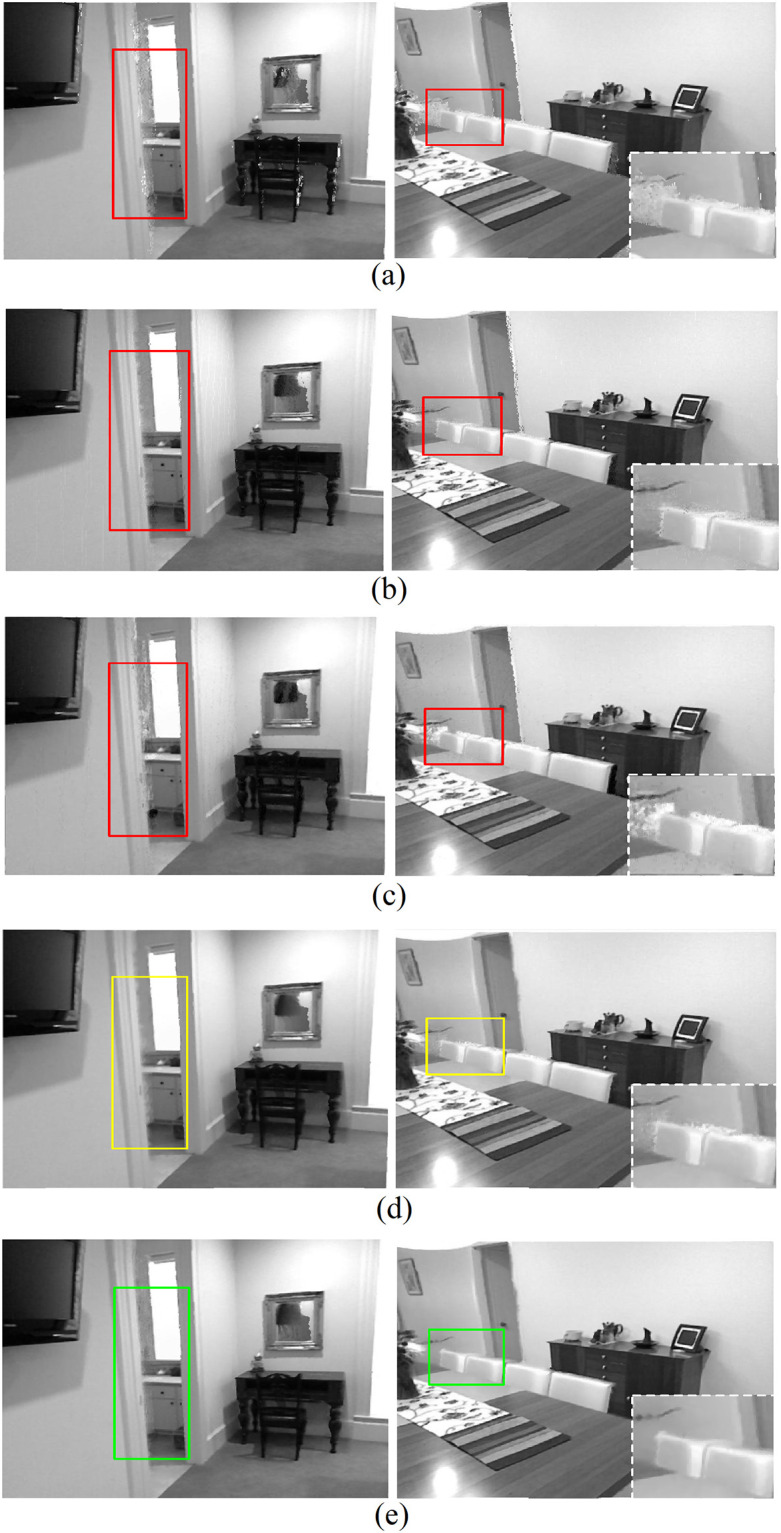
Virtual view images using *NYU Depth* datasets, from top to bottom, A: *EC* B: *SF* C: *GC* D: *Propose1* E: *Propose2*.

The experimental results of the final synthesized virtual view images using test sets from *Middlebury* and *KITTI* are illustrated in Figs 5 and 6 separately. From these results, several observations can be made. First, although deep learning-based generative image inpainting methods have achieved great success recently, they were not specially designed for stereoscopic synthesis and did not perform well in our experiments. Take the areas marked with red rectangles in Figs 5A–5C and 6A–6C, where both the foreground and background textures are mixed on those disoccluison regions of different levels. Second, our proposed network displayed great improvements compared to these conventional generative inpainting methods as shown in the areas of Figs 5D and 6D marked with yellow rectangles. The reason behind this is that our network implemented a progressive structure reconstruction strategy, which also followed the complete DIBR processing pipelines instead of the inpainting-only scheme as the conventional generative inpainting methods did. Some important scene cues in the warped views, such as layer boundaries, can be easily introduced from *ξ*_*c*1_ naturally. Third, the best results were achieved by our method with all proposed additional scene constraints as shown in Figs 5E and 6E, consistent with the previous experimental analysis on the last subsection above. In a word, the proposed method in this paper is suitable for high quality stereoscopic synthesis in the application of 2D-to-3D conversions. Similar conclusions can be drawn in the experimental results shown in Fig 7, where the test sets from the *NYU depth* database are used.

Test sets from both *Middlebury* and *KITTI* have the ground truth for newly generated novel views, so the synthesized virtual view images can further be evaluated by PSNR and SSIM comparisons with the ground truth. Table 2 gives the average PSNR and SSIM comparison results for each database with the best results are highlighted in boldface type. From this table, we can observe that the proposed methods can obtain competitive results compared with the other state-of-the-art generative image inpainting methods. In particular, the experimental results with all proposed scene constraints have the best performances.

**Table 2:** PSNR and SSIM comparisons.

	Database	*EC*	*SF*	*GC*	*Propose1*	*Propose2*
**PSNR(dB)**	*Middlebury*	27.23	28.16	27.19	30.58	**31.21**
	*KITTI*	26.89	27.15	26.74	29.95	**30.81**
**SSIM**	*Middlebury*	0.8912	0.9034	0.8897	0.9223	**0.9317**
	*KITTI*	0.8894	0.8958	0.8842	0.9176	**0.9245**

**Fig 8:**
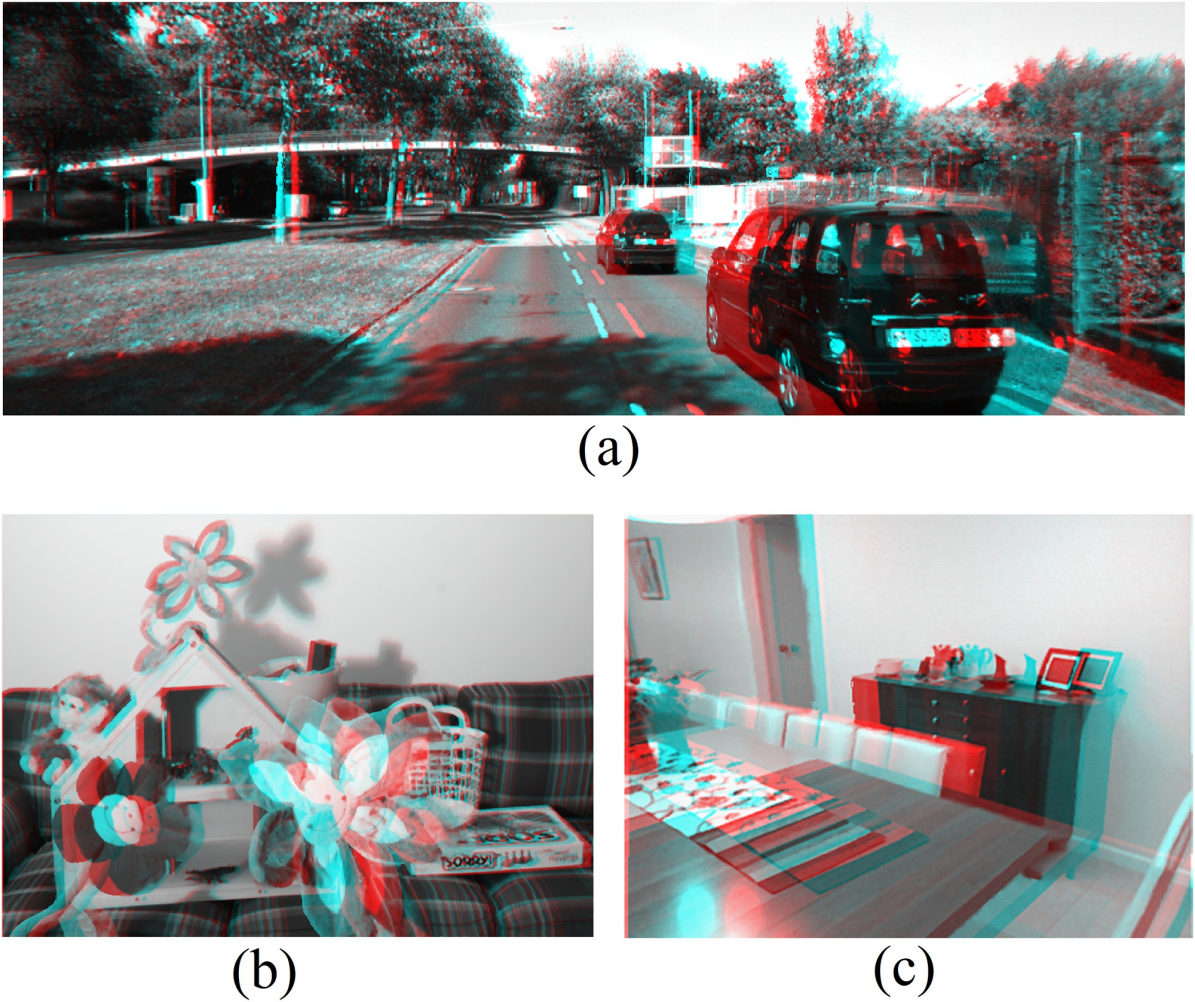
Selected synthesized 3D anaglyph images using test sets from different databases with our proposed approach, A: *KITTI* B: *Middlebury* C: *NUY Depth*.

A human subjective study was also implemented by 15 individuals with normal or correct-to-normal visual acuity. We conducted the test to evaluate the stereoscopic feeling of the final synthesized 3D anaglyph images. The participants watched the synthesized 3D images in a random order and were asked to give a satisfaction score. The scores are from 0 to 5, with higher scores indicating higher stereoscopic feeling. The average scores obtained were used as a measure of the subjective evaluation, as shown in Table 3. Generally speaking, the results are similar to the quantitative ones in Table 2. Our methods performed better in stereoscopic feeling on the synthesized 3D anaglyph images and obtained relatively higher satisfaction scores. Fig 8 shows some examples of the synthesized 3D anaglyph images from the evaluation test sets.

**Table 3:** Results of subjective quality evaluation.

Database	*EC*	*SF*	*GC*	*Propose1*	*Propose2*
*Middlebury*	3.9	4.0	3.9	4.2	**4.3**
*KITTI*	3.8	3.8	3.7	4.1	**4.2**
*NYU Depth*	3.7	3.9	3.8	4.1	**4.2**

## Conclusions

In this paper, we propose a novel learning-based method for stereoscopic view synthesis. In contrast to related existing related methods, we adopt a progressive structure reconstruction strategy instead of direct texture inpainting. In this way, more reasonable scene structures can be added as prior knowledge to gradually improve the disocclusion hole recovery performance. Two special constraints of the synthesized scenes are further exploited for our network to alleviate hallucinated structure mixtures in the warped views. Experimental results demonstrate that the proposed method can obtain competitive results and outperforms other state-of-the-art learning-based stereoscopic synthesis methods in terms of both quantitative metrics and subjective visual qualities, making it more suitable for the 2D-to-3D conversion applications.

## Supporting information

S1 Fig Relevant data underlying the findings described in the experiments of Fig 3. (PPT)

S2 Fig Relevant data underlying the findings described in the experiments of Fig 4. (PPT)

S3 Fig Relevant data underlying the findings described in the experiments of Fig 5. (PPT)

S4 Fig Relevant data underlying the findings described in the experiments of Fig 6. (PPT)

S5 Fig Relevant data underlying the findings described in the experiments of Fig 7. (PPT)

S6 Fig Relevant data underlying the findings described in the experiments of Fig 8. (PPT)
