# Supplementary figures and images for: Stereoscopic view synthesis with progressive structure reconstruction and scene constraints
Source: PLoS One. 2022 Dec 19;17(12):e0279249. doi: 10.1371/journal.pone.0279249 (PMC9762595; doi:10.1371/journal.pone.0279249)

## Slide 1
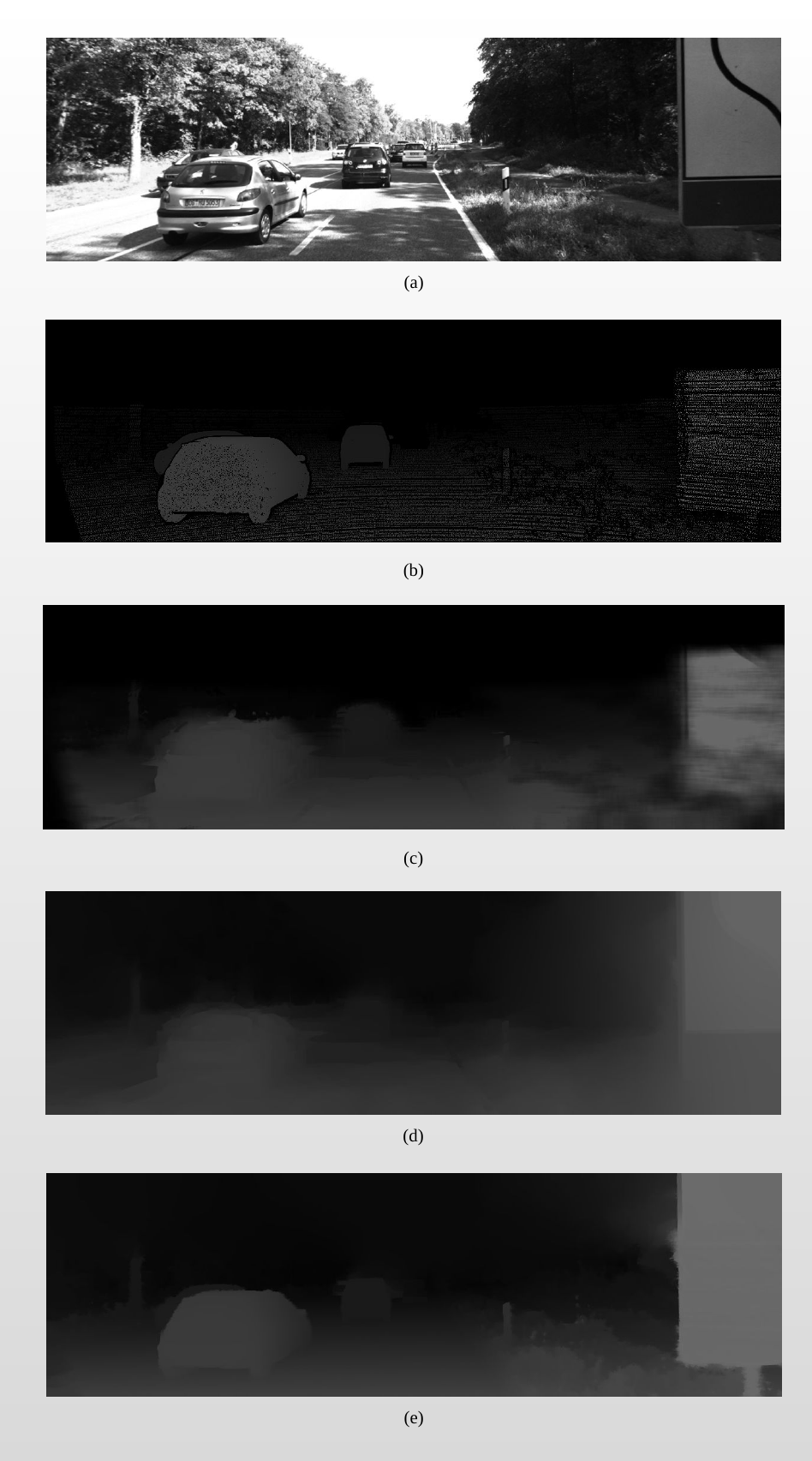

(a)
(b)
(c)
(d)
(e)

Supplement: S1 Fig — (PPT) [file pone.0279249.s001.ppt]

## Slide 1
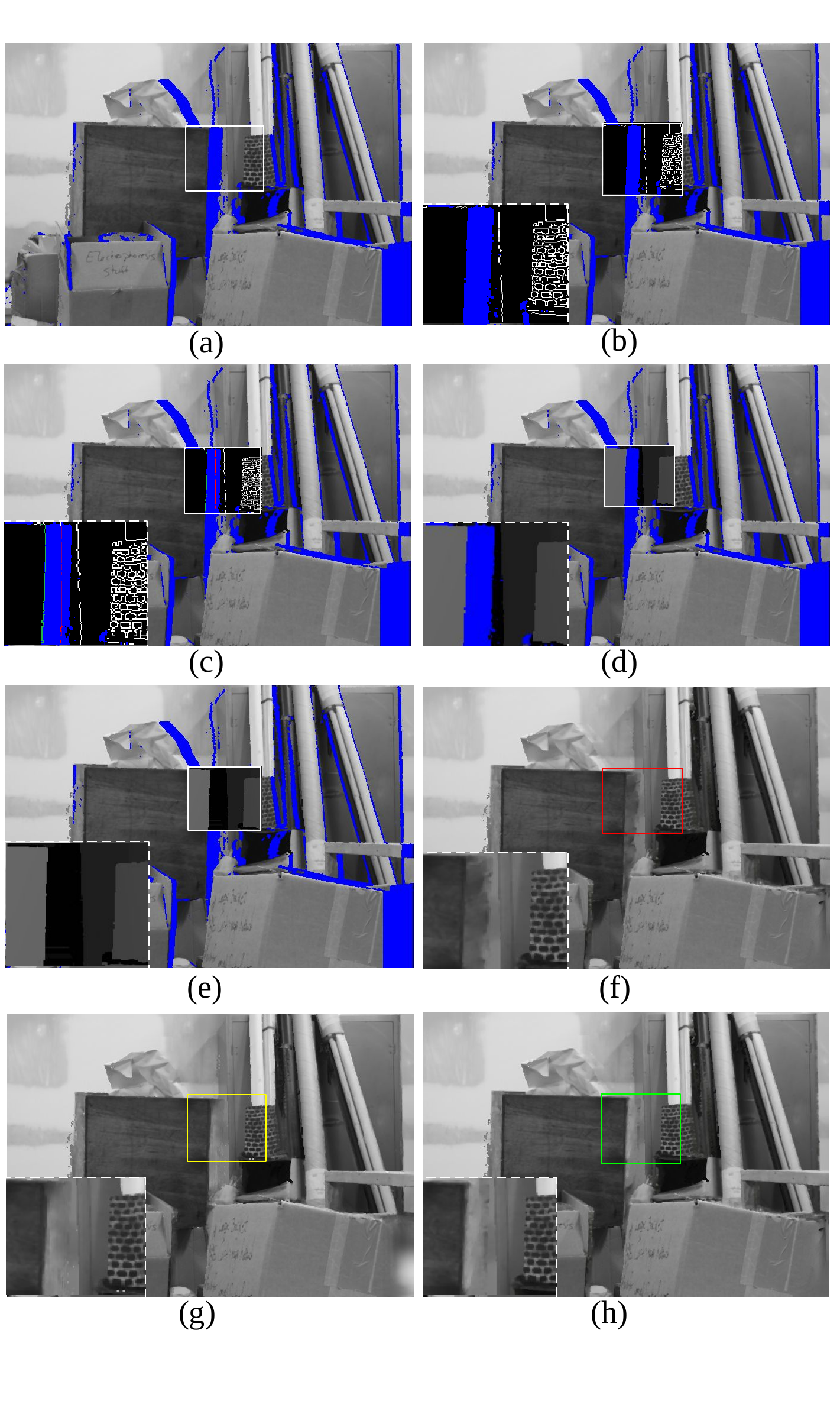

(b)
(a)
(c)
(d)
(e)
(f)
(g)
(h)

Supplement: S2 Fig — (PPT) [file pone.0279249.s002.ppt]

## Slide 1
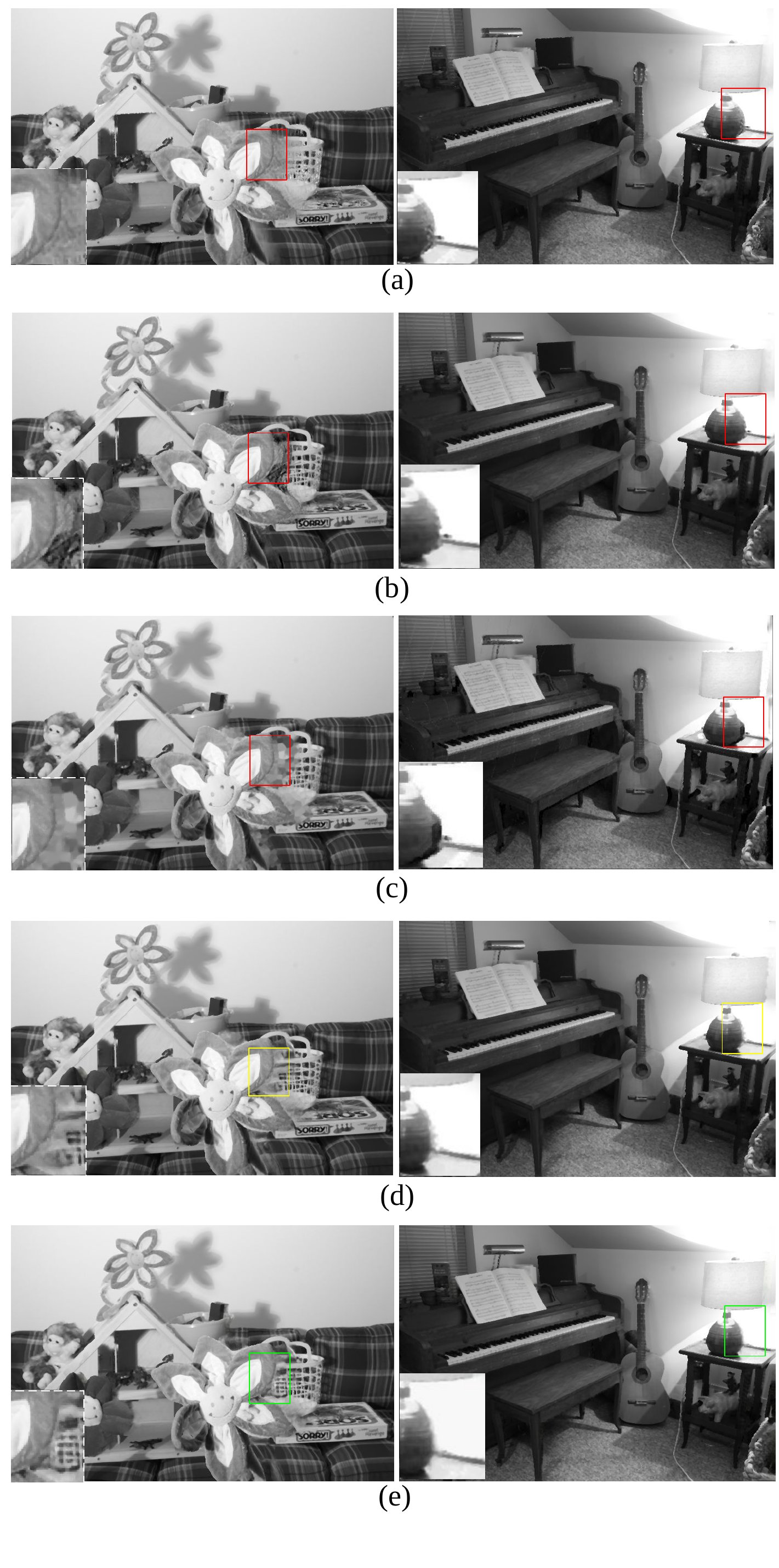

(a)
(b)
(c)
(d)
(e)

Supplement: S3 Fig — (PPT) [file pone.0279249.s003.ppt]

## Slide 1
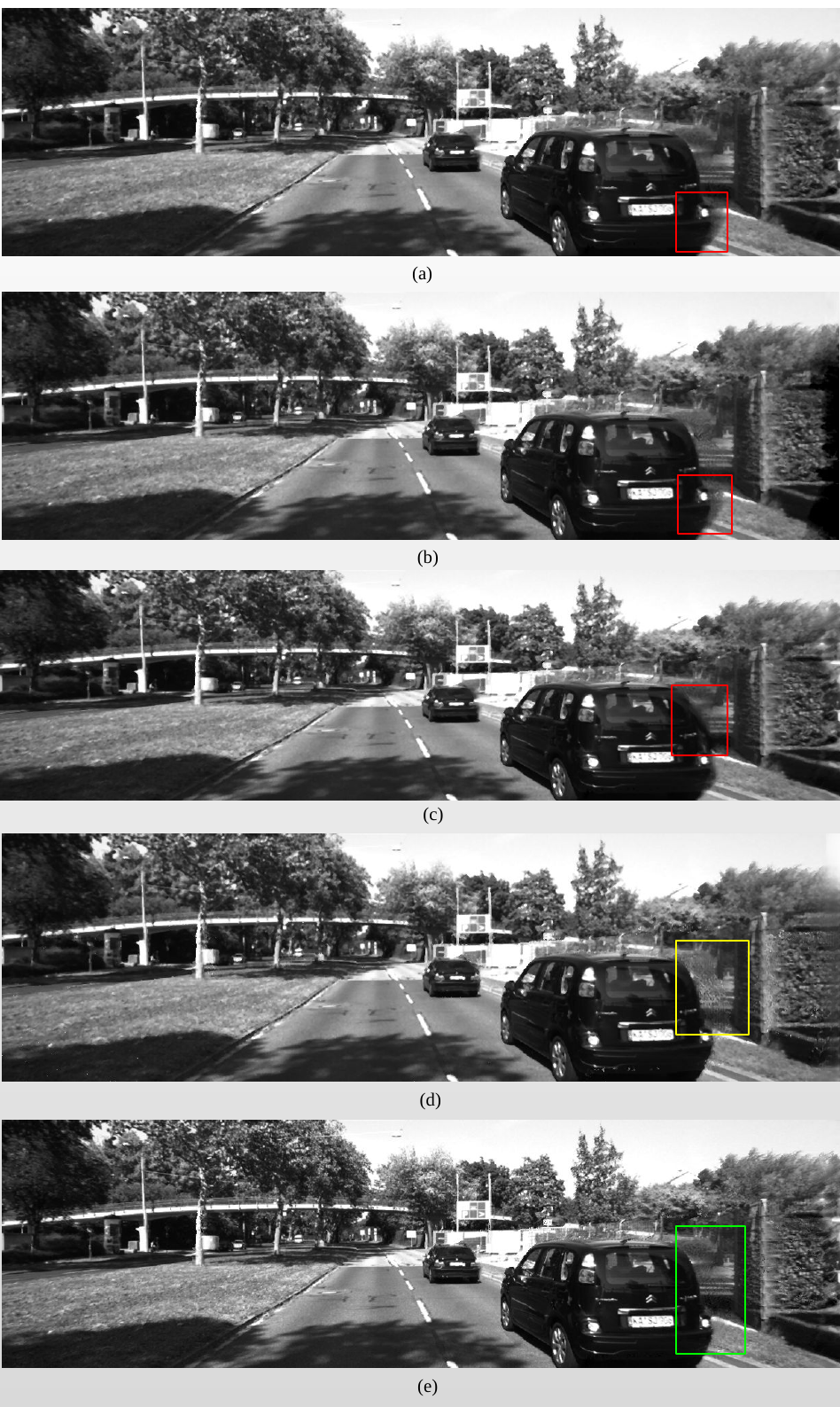

(a)
(b)
(c)
(d)
(e)

Supplement: S4 Fig — (PPT) [file pone.0279249.s004.ppt]

## Slide 1
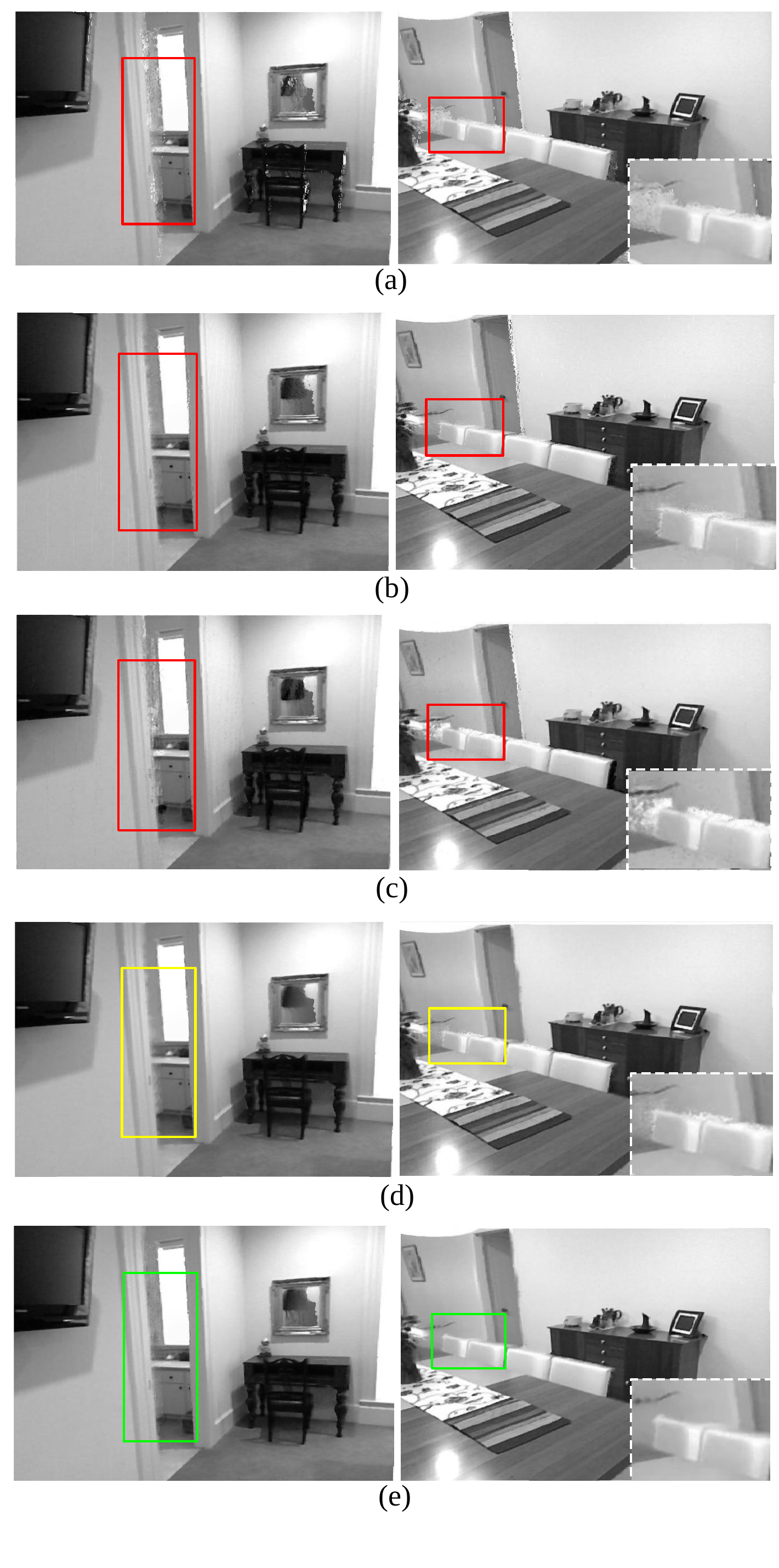

(a)
(b)
(c)
(d)
(e)

Supplement: S5 Fig — (PPT) [file pone.0279249.s005.ppt]

## Slide 1
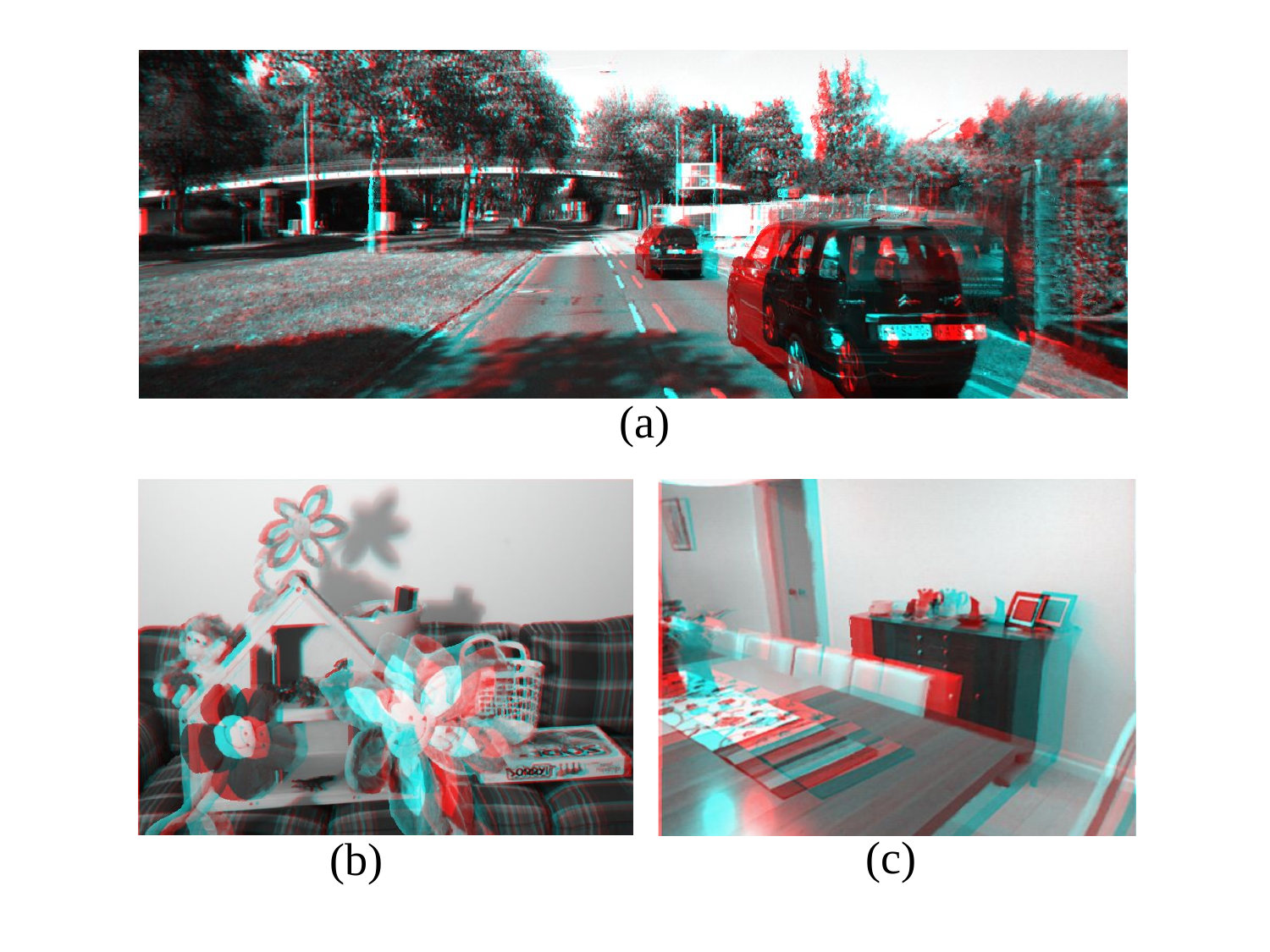

(a)
(c)
(b)

Supplement: S6 Fig — (PPT) [file pone.0279249.s006.ppt]
